# Metallothionein – overexpression as a highly significant prognostic factor in melanoma: a prospective study on 1270 patients

**DOI:** 10.1038/sj.bjc.6603028

**Published:** 2006-02-28

**Authors:** G Weinlich, K Eisendle, E Hassler, M Baltaci, P O Fritsch, B Zelger

**Affiliations:** 1 Clinical Department of Dermatology and Venerology, Innsbruck Medical University, Anichstrasse 35, Innsbruck A-6020, Austria

**Keywords:** metallothionein, melanoma, prognostic factor

## Abstract

Metallothioneins (MT) are ubiquitous, intracellular small proteins with high affinity for heavy metal ions. In the last decades, it was shown that MT overexpression in a variety of cancers is associated with resistance to anticancer drugs and is combined with a poor prognosis. In this prospective study, we examined the role of MT overexpression in melanoma patients as a prognostic factor for progression and survival. Between 1993 and 2004, 3386 patients with primary cutaneous melanoma were investigated by using a monoclonal antibody against MT on routinely fixed, paraffin-embedded tissues. In all, 1270 patients could be followed up for further statistical analysis (Fisher's exact test, Mantel–Haenszel *χ*^2^ test, Kaplan–Meier curves). The MT data of disease-free interval and overall survival were compared univariately and multivariately in Cox regression analysis. Immunohistochemical overexpression of MT in tumour cells of patients with primary melanoma (310 of 1270; 24.4%) was associated with a higher risk for progression (117 of 167; 70.1%) and reduced survival (80 of 110; 72.7%) of the disease (*P*<0.0001). Similarly, Kaplan–Meier curves gave highly significant disadvantages for the MT-positive group. Univariate analysis (relative risk 7.4; 95% confidence interval (CI) 5.2–10.2; *P*<0.0001 for progression; relative risk 7.1; 95% CI 4.7–10.9; *P*<0.0001 for survival), as well as multivariate analysis with other prognostic markers resulted in MT overexpression as a highly significant and independent factor for prognosis in primary melanoma.

Melanoma is one of the most aggressive neoplasms in humans and its incidence is still increasing. As there is still no effective curative treatment in patients with disseminated melanoma, it is important to estimate the risk for progression in the individual patient, preferably at the time point of primary diagnosis. Breslow tumour thickness is still the best prognostic factor for this issue. Notwithstanding, everyone is aware of patients with thick melanoma, who never develop metastases, and patients with ‘low-risk melanomas’ (thinner than 1.0 mm), who will show tumour progression in subsequent years. Therefore, additional factors reliably determining progression and survival would be of great benefit.

Metallothioneins (MT) are a group of ubiquitous, intracellular, low molecular weight proteins (approximately 6.5 kDa) with about 30% consisting of cysteine residues and no aromatic amino acids. They have a specific binding capacity for heavy metal ions of group II such as zinc, copper or cadmium (Nath *et al*, 1988; Miles *et al*, 2000; Nordberg and Nordberg, 2000). To date, four major isoforms have been identified in mammals; the specific functional roles of MT isoforms or molecular interaction are still not precisely known. As far as we know, MT are involved in many physiological and pathophysiological processes such as the intracellular storage, transport and metabolism of heavy metal ions; they regulate essential trace metal homeostasis and play a protective role in heavy metal detoxification reactions (Miles *et al*, 2000; Simpkins, 2000). They can protect cells against UV-/ionic radiation (Hansen *et al*, 1997; Hanada *et al*, 1998; Reeve *et al*, 2000) as well as cytotoxic alkylating agents including chemotherapeutics (Chin *et al*, 1993; Hishikawa *et al*, 1997; Okazaki *et al*, 1998; Sunada *et al*, 2005), modulate oxygen free radicals and nitric oxide and inhibit apoptosis (Tsangaris and Tzortzatou-Stathopoulou, 1998). The synthesis of MT is induced by group II heavy metal ions as well as by endogenous factors such as glucocorticoids, cytokines (interleukin (IL)-1 or IL-6, interferon *γ* (IFN*γ*), tumour necrosis factor *α* (TNF-*α*)) or vitamin D_3_ (Karin *et al*, 1985; Karasawa *et al*, 1987; Schroeder and Cousins, 1990; Sato and Sasaki, 1992; Nishimura *et al*, 2000). There is also some evidence that inflammatory stressors (e.g. heat-shock proteins, reactive oxygen species, endotoxin, IL-1, IL-6) induce MT synthesis, which, subsequently, is strongly chemotactic for inflammatory cells, for example, T cells (Yin *et al*, 2005), and influences angiogenesis (Miyashita and Sato, 2005).

Although MT participate in the carcinogenic process, their use as a potential marker for tumour differentiation or cell proliferation, as well as a marker of poor prognosis remains controversial (Cherian *et al*, 2003; Theocharis *et al*, 2004). In the last decade, several reports disclosed MT overexpression as a useful prognostic factor for tumour progression and drug resistance in a variety of cancers such as ovarian cancer (Surowiak *et al*, 2005), renal cell carcinoma (Mitropoulos *et al*, 2005), breast cancer (Jin *et al*, 2004), non-small-cell lung carcinomas (Dziegiel *et al*, 2004), acute lymphoblastic leukaemia (Sauerbrey *et al*, 1994), pancreatic carcinoma (Ohshio *et al*, 1996) or carcinoma of the gallbladder (Shukla *et al*, 1998). Similar results could be found in smaller retrospective studies in melanoma and nonmelanoma skin cancers (Zelger *et al*, 1993, 1994; Rossen *et al*, 1997; Goldmann *et al*, 1998; Sugita *et al*, 2001). In some other tumours such as colorectal and bladder cancer, MT overexpression was not correlated with increased malignancy (Öfner *et al*, 1994; Jasani and Schmid, 1997).

In 2003, our department published the data of a prospective study on 520 melanoma patients, where we were able to show that MT overexpression is a significant and independent marker for poor prognosis and survival (Weinlich *et al*, 2003). In the present study, we present an update of this prospective cohort 11 years after initiation.

## MATERIALS AND METHODS

### Patients

This prospective study was started in 1993 at the Clinical Department of Dermatology and Venerology, Innsbruck Medical University, Austria. Up to the end of 2004, 3386 patients with a histological diagnosis of cutaneous melanoma were enrolled, and 1270 patients could be evaluated for further statistical analysis. Besides Breslow's tumour thickness and Clark's level, ulceration, location of the primary tumour, age and gender were added for statistical analysis. Measuring end points were the time when a progression (lymph node and/or distant metastasis) was detected and when the patients died due to widespread disease. In the vast majority of our patients (more than 90%), a histopathological verification of the progression was possible by excision of metastases or biopsy of suspicious lesions before initiation of further therapy modalities.

### Histopathological analysis

Formalin-fixed and paraffin-embedded tissues from melanoma patients were routinely stained with haematoxylin and eosin. For immunohistochemical investigations, sections of 4 *μ*m were mounted on chromgel-coated glass slides, deparaffinised in xylene, rehydrated in a graded series of alcohol and rinsed in Tris buffer. Endogenous peroxidase was blocked by means of sodium azide, glucose and glucose-oxidase. The primary monoclonal MT mouse-IgG1 antibody E9 (clone E9; Dako, Denmark), which reacts with both human epitopes of MT I and II isoforms, was applied, followed by a peroxidase-conjugated rabbit-anti-mouse antibody. The enzyme reaction was developed in a freshly prepared solution containing 3-amino-9-ethylcarbazole and 0.01% hydrogen peroxide. Then, the sections were counterstained with haematoxylin, dehydrated, cleared in xylene and mounted with entellan (Evering *et al*, 1990; Jasani and Elmes, 1991). In those cases, where we had multiple blocks of tumour specimens, the immunohistochemical labelling was performed in all of them.

The stained sections were independently assessed by two independent dermatohistopathological observers by an eyeball estimate (EH and MB) without prior knowledge of the clinical data. Generally, a good correlation and reproducibility was obtained by the two observers. In rare cases of disagreement (<5%), both reporting colleges (EH+MB) sat together with the senior author (BZ), who has established this technique previously (Zelger *et al*, 1993; Zelger *et al*, 1994; Weinlich *et al*, 2003), discussed the findings and performed the final evaluation. Only tumour cells staining well above the background level (faint labelling was occasionally observed around strongly positive cells, most likely due to antigen diffusion) were considered to be positive. Reactivity of basal keratocytes, the proliferating epithelium of the follicular bulb and ductal epithelium of eccrine and apocrine glands served as a positive internal control.

**Figure 1:**
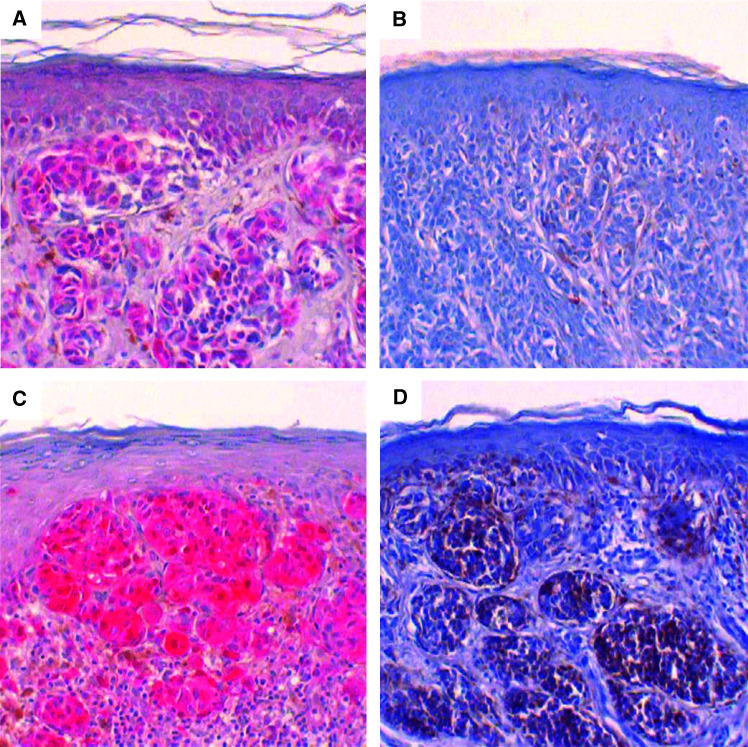
Metallothionein (MT)-positive (**A**+**C**) and MT-negative (**B**+**D**) melanomas with comparable tumour thickness (**A**+**B** 0.6 mm CL III, **C**+**D** 1.0 mm CL IV). The red colour of ACE (in **A**+**C**) indicates positivity, the brown colour (in **D**) represents melanin.

The specimens were scored as negative if there was a complete lack of MT-positive cells, and only a faint hue of reactivity compared with positive internal controls or only a minor component of up to 10% positive tumour cells. Reactivity of more than 10% was interpreted as MT overexpression. The limiting value of 10% was chosen to prevent false-positive results. On the one hand, the physiological proliferation rate of normal tissues as well as the reactivity of various forms of melanocytic naevi are usually smaller than our cutoff level; on the other hand, a counting of cells less than 10%, although perhaps sufficient enough, would be more inexact to measure by an eyeball estimate. In most of the MT-positive cases, the MT monoclonal antibody diffusely labelled the majority of tumour cells (Figure 1).

### Statistical analysis

The ascertained data were statistically analysed using an SPSS statistical software (SPSS for Windows, version 12.0; SPSS Inc., Chicago, IL, USA). The correlation between the immunohistochemical data (absence or presence of MT overexpression) was compared with the clinicopathological data (the occurrence or not of metastasis and/or death due to melanoma) using the two-tailed Fisher's exact test and the Mantel–Haenszel *χ*^2^ test. Kaplan–Meier curves were used to estimate the progression and survival (Kaplan and Meier, 1958). Odds ratios and 95% confidence intervals (CIs) were calculated for all known prognostic factors for progression and survival using Cox regression analysis, assuming proportional hazards in a univariate as well as multivariate approach (Spiegel, 1990). A *P*-value of <0.05 was considered statistically significant in every statistical analysis.

## RESULTS

Between 1993 and 2004, 3386 patients were recruited for this study; 1270 of them could be evaluated for statistical analysis. According to standard guidelines of the Austrian Society of Dermatology and Venerology, the majority of low-risk melanoma patients were followed up by their local dermatologist, but never had dermatological control for follow-up at our department, which was one of the criteria for inclusion into the statistical analysis. In addition, only patients with at least 3 months of observation could enter this study. So out of the 3386 recruited, 1226 patients had to be dropped, because they were lost for follow-up (observation <3 months). The majority of the lost patients had low-risk melanomas <1.0 mm or *in situ* melanomas (*n*=1167, 95%). An additional 890 patients had to be dropped out for the statistical analysis (*in situ* melanomas). Within the rest of the group of 1270 statistically evaluable patients (>*in situ*, ⩾3 months, complete data set), males and females were nearly balanced (51%/49%), and the median age at the time of excision was 54 years (range 7–95 years). Breslow tumour thickness varied between 0.12 mm and a tumour thickness of 30 mm (median value 0.7 mm, mean 1.3 mm). Table 1 gives the characteristics of the 3386 patients included in the data set and of those 1270 eligible for further analysis.

In 11 years of recruitment (median observation time 32 months), 167 of 1270 patients (13.1%) showed a progression of their disease, and the median time to progression was 18.0 months (mean value 26.0 months). In all, 110 of these patients (8.7%) died because of the metastatic disease of melanoma. The median time between detection of the first metastasis to death was 9.0 months (mean 15.3 months), and the median time from primary diagnosis of melanoma to death was 26.5 months (mean 35.1 months). None of our patients with a tumour thickness <0.5 mm developed metastases.

The majority of patients who showed progression, that is, 117 of 167 (70.1%), and from those who died due to metastasis, that is, 80 of 110 (72.7%), showed an MT-overexpression of their primary melanoma (*P*<0.001, *χ*^2^ test; Fisher's exact test) (Table 2). Even in patients with low-risk melanomas <1.0 mm (846 of 1270), the majority of those with progression, that is, 21 of 32 (65.6%, *χ*^2^=63.9; df=1; *P*<0.001), and those who died, that is, 11 of 15 (73.3%, *χ*^2^=39.0; df=1; *P*<0.001), showed statistically significant MT overexpression in their primary melanoma.

**Figure 2:**
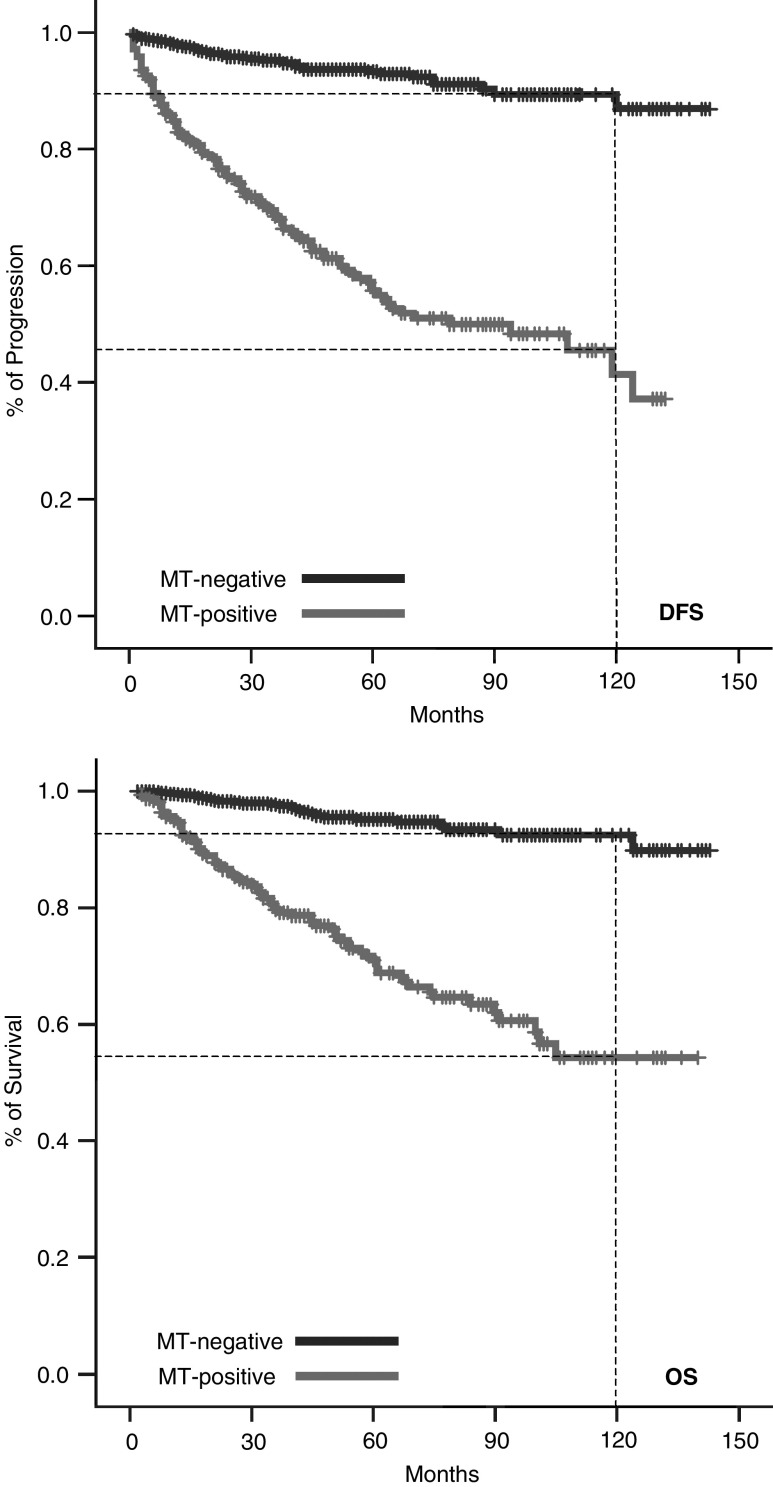
Kaplan–Meier estimates for progression and survival in melanoma patients.

**Figure 3:**
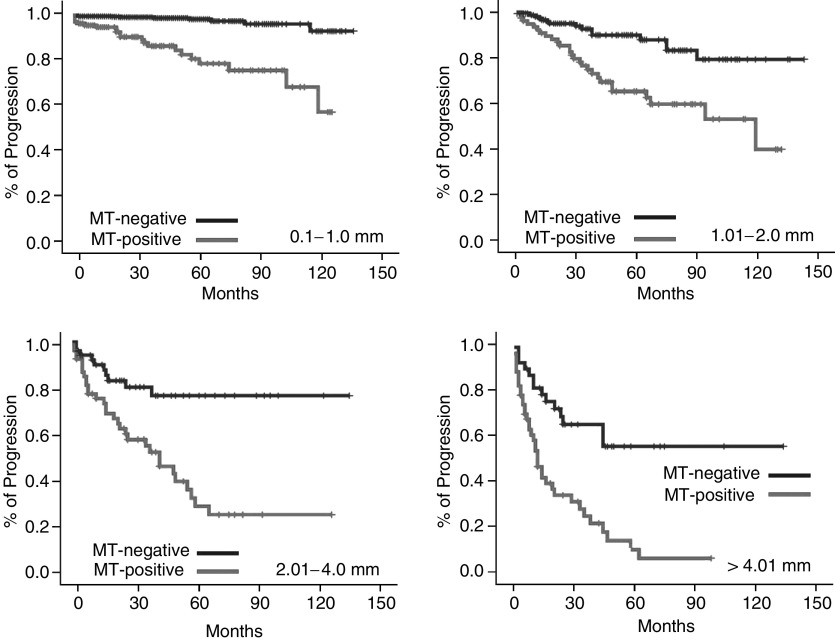
Kaplan–Meier curves for progression in melanoma patients in the different groups of Breslow tumour thickness.

Figure 2 illustrates the progression and survival of melanoma patients over a time period of 150 months estimated by the Kaplan–Meier method. A total of 55.9% of the MT-positive group, but only 10.9% of the MT-negative group developed metastasis after 10 years of observation (*P*<0.0001); 44.1% of MT-positive patients died within a period of 120 months due to progression of melanoma, but only 6.8% of patients in the MT-negative group (*P*<0.0001). According to the tumour node metastasis (TNM) classification of the American Joint Committee on Cancer 2001, we subdivided our patients into four groups (<1.0 mm, 1.01–2.0 mm, 2.01–4.0 mm, >4.01 mm) with similar results. Even in the group of low-risk melanomas thinner than 1.0 mm, the prognosis for the MT-positive group was worse in comparison with the MT-negative group. Figure 3 illustrates the Kaplan–Meier curves for the progress-free interval; analogous data for survival are not shown.

As the percentage of MT-positive melanomas was increasing with higher Clark level and Breslow tumour thickness, we tried to find out in a next step if MT overexpression is an independent prognostic marker or just a parameter correlated with increasing invasion level or tumour thickness. First, in a univariate Cox proportional hazard model for disease progression, we found a high significance of MT overexpression with an expected risk of 7.4 (95% CI 5.3–10.2). Metallothionein overexpression as well as all groups of Breslow tumour thickness had *P*-values <0.001 in the univariate analysis (Table 3). The patient's data were also calculated according to survival with similar results (expected risk 7.2, 95% CI 4.7–10.9; *P*<0.001).

Then, we adjusted the data of our patients according to other prognostic factors such as Breslow tumour thickness, Clark level, ulceration, tumour localisation, median age, gender and MT overexpression in a multivariate Cox regression analysis to prove the independence of these factors. The strongest significant parameters for progression were tumour thickness and MT overexpression; only Clark level V reached a significance of *P*<0.05, and ulceration was not significant in our cohort (Table 4). In the Cox regression model for survival, we found similar results with two highly significant and independent parameters: MT overexpression and Breslow tumour thickness.

For a further calculation, we adapted the regression analysis with Breslow tumour thickness and age as continuous variables (table not shown). In this model, tumour thickness (expected risk 1.13; 95% CI 1.1–1.2; *P*<0.001) and again MT overexpression (expected risk 4.2; 95% CI 2.9–5.9; *P*<0.001) showed significant results; Clark level IV (expected risk 5.9, 95% CI 1.8–19.3; *P*=0.003), Clark level V (expected risk 11.4, 95% CI 3.2–40.1; *P*<0.001), ulceration (expected risk 1.5; 95% CI 1.0–2.2; *P*=0.035) as well as age (expected risk 1.0, 95% CI 1.01–1.03; *P*=0.001) were significant too. Analogous results could be found when we calculated the same data in the analysis for survival, with only a small difference; the localisation also reached a *P*-value <0.02 (expected risk 1.7, 95% CI 1.1–2.6).

## DISCUSSION

Although there is still no effective treatment for disseminated melanoma (stage IV), it is of great interest to estimate the risk for a possible progression as early as possible, at the time point of the primary diagnosis of melanoma. Up to now, Breslow tumour thickness is still the best prognostic marker in primary melanoma. Over the preceding years, a wide variety of different prognostic factors have been investigated: Clark level, tumour stage, growth phase, ulceration, mitotic counts or tumour-infiltrating lymphocytes and many others. Most of these other prognostic markers often derive their predictive value because of a direct or secondary correlation with tumour thickness.

In this prospective study on 1270 melanoma patients with a long time of follow-up, our statistical analysis was able to document that MT overexpression is a potent, highly significant and tumour thickness-independent factor, which helps to assess and calculate the risk for subsequent progression. This could be shown by several statistical methods like *χ*^2^ test, Fisher's exact test, Kaplan–Meier curves, as well as uni- and multivariate Cox regression analysis. Our data are highly significant in all used test systems, although 2116 out of 3386 initially included patients had to be dropped out, 95% of them with melanomas <1.0 mm, *in situ* melanomas or because of too short a time of observation, due to our strict inclusion criteria.

Our results can be interpreted from different aspects: on the one hand, MT-negative melanoma thinner than 1.0 mm practically never cause any further problems. There were only three cases of metastasis from an MT-negative melanoma thinner than 1.0 mm (three out of 1067 MT-negative cases, 0.28%); two of them had distinct histological signs of regression. In our cohort, none of the patients with tumours <0.5 mm (591 patients with 54/9.1% MT positive) developed metastasis in the subsequent years. This may assure such patients of their good long-term outcome. So it should be deliberated if MT-negative patients with melanomas thinner than 1.0 mm could be controlled more generously without ultrasound- or X-ray-staging examinations. On the other hand, MT-positive melanoma thinner than 1.0 mm are at a higher risk of developing metastasis and succumbing to their neoplastic disease. In our study population, 5.3% of the patients in this ‘low-risk’ group (nine out of 170 MT-positive melanomas <1.0 mm) showed a progression of their disease; their relative risk is roughly comparable to MT-negative melanoma with a thickness of 2.1–4 mm. This may be used to more carefully follow up these patients and/or probably even serve as a tool to indicate and perform sentinel lymph node biopsy. Moreover, this group of patients could probably profit from adjuvant treatment.

Metallothionein overexpression probably has an additional value. In stage IV melanoma patients, anticancer drugs, as well as irradiation therapy, are known to often show only a humbled rate of clinical responses. These therapeutic failures may partially be related to an enhanced MT overexpression in tumour cells, although the involvement of MT in conferring resistance to chemotherapeutics still remains under discussion (Chin *et al*, 1993; Hishikawa *et al*, 1997; Okazaki *et al*, 1998; Cherian *et al*, 2003). As a variety of endogenous factors (e.g. glucocorticosteroids, ILs, IFN*γ*, TNF-*α*) are involved in the induction of the synthesis of intracellular MT, one may suggest that this may lead to an overprotection of tumour cells against apoptosis, and, on the other hand, supporting the metastatic behaviour of the tumour (Karin *et al*, 1985; Karasawa *et al*, 1987; Nath *et al*, 1988; Schroeder and Cousins, 1990; Sato and Sasaki, 1992; Tsangaris and Tzortzatou-Stathopoulou, 1998; Miles *et al*, 2000; Nishimura *et al*, 2000).

In summary, our data confirm previous results of retrospective and much smaller studies in melanoma, outlining that MT overexpression is a useful and elegant tool for prognostication (Zelger *et al*, 1993; Goldmann *et al*, 1998; Sugita *et al*, 2001). This marker is highly significant and independent of tumour thickness and already predictive in low-risk melanomas thinner than 1.0 mm. These investigations by immunohistochemical labelling on archival paraffin material are easy to assess and perform in routine pathology and dermatopathology laboratories and the costs are limited.

## Figures and Tables

**Table 1:** Characteristics of melanoma patients 1993–2004

	**n=3386**	**n=1270**
**Number of patients**	**(all patients recruited 1993–2004)**	**(>*in situ*, ⩾3 months observation)**
*Observation time (months)*		
Range	0–143	3–143
Mean	26.3	42.5
Median	13.0	32.0
		
*Age (years)*		
Range	5–100	7–95
Mean	55.0	53.2
Median	57.0	54.0
		
*Sex*		
Male	1589 (46.9%)	649 (51.1%)
Female	1797 (53.1%)	621 (48.9%)
		
*Localisation*		
TANS	2228 (65.8%)	772 (60.8%)
Not TANS	1158 (34.2%)	498 (39.2%)
		
*Ulceration*		
Present	157 (4.6%)	123 (9.7%)
Absent	3229 (95.4%)	1147 (90.3%)
		
*Tumour thickness (mm)*		
*In situ*	1613 (47.6%)	
<1.0	1291 (38.1%)	846 (66.6%)
1.01–2.0	263 (7.8%)	236 (18.6%)
2.01–4.0	124 (3.7%)	104 (8.2%)
>4.01	95 (2.8%)	84 (6.6%)
		
*Invasion level*		
CL I	1613 (47.6%)	
CL II	257 (7.6%)	176 (13.9%)
CL III	1084 (32.0%)	736 (71.8%)
CL IV	376 (11.1%)	313 (24.6%)
CL V	56 (1.7%)	45 (3.5%)

CL=Clark level; TANS=thorax, upper arm, neck, scalp.

**Table 2:** Characteristics of MT overexpression in primary melanoma

		**MT-positive**		**MT-negative**	
Number of melanoma	1270	310	24.40%	960	75.60%
*Tumour thickness (mm)*					
<1.0	846	131	15.50%	715	84.50%
1.01–2.0	236	78	33.10%	158	66.90%
2.01–4.0	104	53	51.00%	51	49.00%
>4.01	84	48	57.10%	36	42.90%
					
					
Progression (*n*=167, 13.1%) (*χ*^2^=217.2, df=1, *P*<0.001)		117	70.10%	50	29.90%
				
Death (*n*=110, 8.7%) (*χ*^2^=152.4, df=1, *P*<0.001)		80	72.70%	30	27.30%

MT=metallothionein.

**Table 3:** Univariate Cox proportional hazard model for disease progression and survival (tumour thickness/MT overexpression)

	**Progression**			**Survival**		
***n*=1270**	**Relative risk**	**CI 95%**	***P*-value**	**Relative risk**	**CI 95%**	***P*-value**
MT overexpression	7.36	5.28–10.25	<0.001	7.16	4.71–10.9	<0.001

*Tumour thickness (mm)*						
<1.0	1			1		
1.01–2.0	4.24	2.68–6.72	<0.001	5.44	2.9–10.19	<0.001
2.01–4.0	11.1	6.99–17.62	<0.001	11.56	6.03–22.16	<0.001
>4.01	25.25	16.2–39.34	<0.001	35.06	19.49–63.07	<0.001

CI=confidence interval; CL=Clark level; MT=metallothionein.

**Table 4:** Multivariate Cox regression analysis results for disease progression and survival in melanoma patients

	**Progression**			**Survival**		
***n*=1270**	**Relative risk**	**CI 95%**	***P*-value**	**Relative risk**	**CI 95%**	***P*-value**
MT overexpression	3.94	2.77–5.6	<0.001	3.49	2.25–5.43	<0.001
*Tumour thickness (mm)*						
<1.0	1			1		
1.01–2.0	1.97	1.14–3.41	0.015	2.55	1.24–5.25	0.011
2.01–4.0	3.46	1.91–6.28	< 0.001	3.64	1.63–8.13	0.002
>4.01	6.58	3.49–12.43	< 0.001	8.83	3.93–19.83	<0.001
						
*Clark level*						
CL II	1			1		
CL III	2.11	0.64–6.95	0.22	3.09	0.40–23.57	0.277
CL IV	3.29	0.94–11.50	0.062	4.94	0.61–39.79	0.133
CL V	5.75	1.51–21.90	0.01	8.36	0.98–71.55	0.053
						
*Ulceration*						
Present	1.39	0.96–2.03	0.079	1.46	0.93–2.28	0.094
						
*Age (years)*						
>54	1.62	1.15–2.27	0.05	1.52	1.00–2.30	0.05
						
*Gender*						
Female	0.84	0.60–1.16	0.279	1.04	0.69–1.54	0.862
						
*Location*						
TANS	0.88	0.64–1.22	0.443	1.48	0.99–2.22	0.059

CI=confidence interval; CL=Clark level; MT=metallothionein; TANS=thorax, upper arm, neck, scalp.
